# Angiopoietin-2 adsorption attempt with the cytokine adsorber cytosorb in critically ill patients

**DOI:** 10.1038/s41598-025-21215-y

**Published:** 2025-10-01

**Authors:** Helen Graf, Caroline Gräfe, Michael Paal, Katarina Habler, Alice Ewert, Wolfgang Wilfert, Uwe Liebchen, Martin Bender, Danilo Hackner, Christina Scharf

**Affiliations:** 1 Department of Anesthesiology, University Hospital, LMU Munich, Marchioninistrasse 15, 81377 Munich, Germany; 2 Institute of Laboratory Medicine, LMU University Hospital, LMU Munich, Munich, Germany

**Keywords:** Angiopoietin-2 (Ang-2), Endothelial dysfunction, Sepsis, Rhabdomyolysis, Cytosorb, Medical research, Nephrology

## Abstract

Angiopoietin 2 (Ang-2) is a growth factor in angiogenesis, causing pericyte migration and vascular regression, leading to increased vascular permeability. Inflammatory mediators, hypoxia, hyperglycaemia and cancer stimulate its secretion. Particularly in sepsis, high Ang-2 levels appear to be a prognostic marker for morbidity and mortality in the critically ill. As an important trigger for endothelial damage one therapeutic option in these patients might be the removal of Ang-2. The Cyto-SOLVE trial included patients with septic shock or rhabdomyolsis treated with the adsorber Cytosorb (CS) (included in continuous kidney replacement therapy) for the removal of cytokines and myoglobin. Ang-2 was measured in the patient´s blood and pre- and post-CS at defined time points (ten minutes, one, three, six, and twelve hours after initiation). Ang-2 extracorporeal clearances (ml/min) were calculated with: $\:\left(bloodflow*\left(1-hematocrit\right)\right)*\left(\frac{concentration\:\left(pre-post\right)}{concentration\:\left(pre\right)}\right).$ *U*-Test was used to compare different subgroups. 26 patients were included (17 with rhabdomyolsis and 9 with septic shock, median Ang-2 concentration prior to CS-application: 21,653 pg/ml). We observed a low Ang-2 adsorption in the first hour of CS application, resulting in a median Ang-2 extracorporeal clearance of 8.2 ml/min and 1.7 ml/min after 10 min and 1 h, respectively. There was no significant change in Ang-2 plasma concentrations at any time point. No significant difference in Ang-2 levels was observed between patients with septic shock (median: 31,916 pg/ml) and rhabdomyolysis (median 18,379 pg/ml, *p* = 0.241). The Cytosorb adsorber does not decrease Ang-2 concentrations by adsorption. Therefore, Ang-2 can be considered a reliable biomarker for endothelial dysfunction even in the presence of Cytosorb therapy in critically ill patients. Further studies should be conducted to determine whether the removal of Ang-2 could be beneficial when utilizing an appropriate device.

## Introduction

Angiopoietin-2 (Ang-2) belongs to a family of growth factors (Angiopoietin-1–4) and is a glycoprotein with a molecular weight of approximately 57–70 kDa, depending on its glycosylation and present molecular structure^1–3^. It acts through tyrosine kinase receptors and plays a crucial role in angiogenesis^4^. Ang-2 and Ang-1 both bind to the Tie2-receptor in endothelial cells. Ang-1 acts as an agonist through autophosphorylation, while Ang-2 is mainly an antagonist at the Tie2-receptor, although it can have an agonistic effect at very high concentrations (> 800 ng/ml)^5,6^. In this way, they fulfil opposing functions in angiogenesis, while Ang-1 promotes vascular stabilization, whereas Ang-2 causes the migration of pericytes, vessel regression and disconnection of endothelium leading to an increased vascular permeability^1,4^.

Since inflammatory mediators, hypoxia, hyperglycemia, and cancer stimulate the rapid autocrine secretion of Ang-2, several diseases are associated with high levels of Ang-2^4,7^. Especially in septic patients, high Ang-2 concentrations reflect the clinical picture of endothelial disorders, capillary leak, hypovolemia and vasoplegia^8,9^. Several studies even describe Ang-2 as a prognostic marker for morbidity and mortality in patients with sepsis^8,10–13^.

Furthermore, patients with acute kidney injury (AKI) with and without sepsis develop high levels of Ang-2^14–16^. For example, Robinson-Cohen et al. described that high plasma concentrations of Ang-1 and Ang-2 are associated with AKI in critically ill patients, independent of inflammation^17^. Furthermore, Liu et al. reported Ang-2 as a predictor of AKI in patients after acute myocardial infarction^18^. In addition, Ang-2 also seems to be a predictor for the need of renal replacement therapy (RRT) in patients with chronic kidney disease^19^.

Due to the high clinical relevance, the angiopoietin/tie-pathway became an interesting point of application for several therapeutic approaches^20,21^. Ang-2 has become a target of specific antiangiogenic therapies, particular in cancer therapy and macular disease^22,23^. Since Ang-2 appears to be an important trigger for endothelial damage, especially in critically ill patients, one therapeutic option could be the removal of Ang-2. Lovric et al.. demonstrated a successful removal by plasma exchange in critically ill patients with thrombotic microangiopathy and anti-glomerular basement membrane disease^24^.

The cytokine adsorber Cytosorb (CS), which eliminates molecules up to a size of 60 kDa and is approved for the removal of cytokines, bilirubin, myoglobin, ticagrelor and rivaroxaban, is widely used for these indications^25,26^. As there are references that the adsorption spectrum might be beyond the above mentioned molecular weight, an extracorporeal removal of Ang-2 (57–70 kDa) might be as well possible^27,28^. The primary question of interest was whether the adsorber Cytosorb could efficiently remove Ang-2. Secondary questions included whether there were significant differences in Ang-2 concentrations between the subgroups and whether there was an improvement in clinical parameters due to the potential removal of Ang-2. Given that Ang-2 leads to increased vascular permeability, fluid balance and catecholamine dosages appeared to be suitable parameters for investigation.

## Methods

### Study setting

This was a post-hoc analysis of a single-center, prospective, observational trial including patients with Cytosorb application for the adsorption of cytokines in patients with septic shock or the adsorption of myoglobin in patients with severe rhabdomyolysis. The patients were included between May 2021 and April 2023 during their stay at two intensive care units (ICU) at the LMU hospital in Munich. The local institutional review board approved the study (registration number 21-0236). The study was registered in clinicaltrials.gov (NCT04913298; 04/06/2021). Ethical approval was obtained from the ethical review committee of the Ludwig-Maximilians-Universität (registration number 428-12), also the study was performed in accordance with relevant named guidelines and regulations. Prior to inclusion in the study, written informed consent was obtained from patients or their legal representatives as approved by the review board.

### Study population

Adult patients (≥ 18 years) with the need for continuous RRT due to anuric/oliguric acute kidney injury, diagnosed according to the KDIGO consensus criteria, and CS-application, were included. In addition, patients had to be diagnosed with septic shock combined with hyperinflammation marked out by an IL-6 concentration > 500 pg/ml in patients´ blood or severe rhabdomyolysis with plasma myoglobin levels > 5000 ng/ml as there is no general definiton of rhabdomyolysis. As mentioned above, the exclusion criterion was no consent from the patient or their legal representatives to participate in the study. The indication of CS application was at the discretion of the attending physician and independent of the study.

### Blood sampling

CS was installed post-dialyzer into the Fresenius MultiFiltrate circuit (MultiFiltrate CVVHD (Continuous veno-venous hemodialysis/hemodiafiltration) CiCa^®^ Ultraflux^®^ AV 1000 S or CVVHDF MultiBic^®^ Ultraflux^®^ AV 600 S, post-dilution). Whole blood (EDTA) was taken at the extracorporeal circuit directly before the cartridge (= pre-CS) and directly after the cartridge (= post-CS) at defined time points. The sampling times were 10 min after the start of CS treatment, and 1, 3, 6, and 12 h after initiation. Furthermore, Ang-2 plasma levels were measured shortly before the initiation of CS and after 6 and 12 h. Plasma was obtained by centrifuging at 2500 G for 10 min at room temperature, then securely stored at − 80 °C in safe-lock tubes until laboratory analysis.

### Laboratory measurements

The laboratory parameters myoglobin and IL-6 were measured in plasma using the standard clinical chemistry modular analyzer Cobas^®^ 8000 (Roche Diagnostics, Mannheim, Germany) at the Institute of Laboratory Medicine. Both markers are available on a 24/7 basis, with a turnaround time of approximately one hour. Lactate was quantified from whole blood with the ABL800 Flex blood gas analyzer (Radiometer, Krefeld, Germany). Ang-2 was measured from a separate aliquot stored at − 80 °C and immediately analyzed after a single thaw. It was quantified with the human Angiopoietin-2 Quantikine^™^ ELISA Kit from R&D Systems (Biotechne, Mineapolis, MN, USA) according to the manufacturer instructions, with plasma samples diluted 10-fold in the provided calibrator diluent. The ELISA kit is designed to quantify naturally occurring human Ang-2, that forms disulfide-linked dimers, trimers, tetramers, and pentamers.

### Data collection

For data evaluation, demographic data and clinical data as well as laboratory variables were collected from the laboratory and patient information system. Laboratory parameters like CRP, creatinine, urea, total bilirubin, LDH, myoglobin and IL-6, were measured shortly before CS initiation in the clinical routine.

### Statistical analysis

The statistical analysis was performed using IBM SPSS statistics (Version 29.0. IBM Corp., Armonk, NY, USA). A paired *T-test* with associated samples was used to compare the concentrations pre- and post-CS after testing a normal distribution of the studied parameters (Shapiro–Wilk-test). For variables without a normal distribution, the Wilcoxon-Test was performed. Subgroup analysis was performed comparing patients with septic shock and rhabdomyolysis. *U-test* and Fisher´s exact test was used to differentiate the subgroups. The Ang-2 extracorporeal clearances of CS were calculated with:

$$\:Extracorporeal\:Clearance\:\left(\frac{ml}{min}\right)=\left(bloodflow\left(\frac{ml}{min}\right)*\left(1-hematocrit\right)\right)*\left(\frac{concentration\:\left(pre-post\right)}{concentration\:\left(pre\right)}\right)\:$$

The Ang-2 concentration was correlated with clinical (norepinephrine dosage, fluid balance and 28-days mortality) and laboratory parameters (myoglobin, IL-6) using the Spearman correlation coefficient and point-biserial correlation. A *p*-value of *p* < 0.01 (Bonferroni correction for multiple testing, alpha level = 0.05) was considered statistically significant.

## Results

### Demographic and clinical data

A total of 26 patients were included in the study. The indication for CS treatment was septic shock with hyperinflammation (34.6%) or rhabdomyolysis (65.4%). The main diagnosis that led to admission to the ICU were diverse. In descending order in patients with septic shock: sepsis (44.4%), acute respiratory distress syndrome (ARDS) (22.2%), one patient after liver transplantation, one patient with intestinal ischemia and one patient after resuscitation. In descending order in patients with rhabdomyolysis: liver or lung transplantation (29.4%), ARDS (29.4%), major vascular surgery (17.6%), one patient with polytrauma, one patient after resuscitation and one patient with pericardial tamponade. The majority of the patients (77%) were already undergoing dialysis before the start of CS (66.6% of the patients with sepsis, 87.5% of the patients with rhabdomyolysis). There was no death during the sampling period. In six patients the treatment with CS had to be interrupted before the end of the 12 h period due to a blocked filter (*n* = 4), citrate accumulation with switch to CVVHDF (*n* = 1) or emergency surgery (*n* = 1).

92.3% of the study population still required dialysis 28 days after CS treatment or until death. Table 1 displays detailed patient characteristics in the whole cohort as well as separated for both subgroups.

**Table 1:** Patient characteristics and laboratory measurements of patients with rhabdomyolysis and septic shock.

	Whole cohort	Patients with septic shock	Patients with rhabdomyolysis	*p*-value
	*n* (%) or median [IQR]			
Patient characteristics				
Number of patients	26	9	17	
Age (years)	54 [41; 63]	56 [48; 64]	51 [39; 62]	0.367
Gender: male/female	20 (77)/6 (23)	7 (78)/2 (22)	13 (76)/4 (24)	0.668
Weight (kg)	80 [74; 94]	75 [70; 80]	80 [75; 110]	0.263
Height (m)	1.79 [1.72; 1.84]	1.8 [1.72; 1.85]	1.77 [1.72; 1.82]	0.833
BMI (kg/m^2^)	25 [22; 30]	25 [23; 26]	26 [22; 33]	0.241
28-days mortality	15 (58)	7 (78)	8 (47)	0.138
SAPS-II at the day of treatment	72 [61; 80]	62 [59; 72]	76 [62; 82]	0.065
Renal replacement therapy				
CVVHD (CiCa^®^)/ CVVHDF (MultiBic^®^, post-dilution)	17 (65)/9 (35)	3 (33)/6 (67)	14 (82)/3 (18)	**0.02**
Blood flow (ml/min)	100 [100; 200]	150 [100; 250]	100 [100; 200]	0.241
Dialysate flow (ml/h)	2000 [2000; 2000]	2000 [2000; 2500]	2000 [2000; 2000]	0.916
Substitute flow (if CVVHDF)	1900 [0; 2000]	1800 [0; 2000]	1001 [1000; 2250]	0.6
Ultrafiltration (ml/h)	50 [0; 125]	0 [0; 135]	75 [0; 127.5]	0.187
Urine output during treatment	0 [0; 20]	0 [0; 50]	0 [0; 20]	0.491
Time on dialysis before CS (h)	48.2 [15,3; 202,6]	11.8 [0; 252]	69.3 [30,3; 227,3]	0.077
Time between ICU admission and start CS (h)	133.3 [25.5; 308]	35 [13.7; 486]	162 [44.3; 471.5]	0.225
Laboratory parameters shortly before initiation of cytosorb				
Angiopoietin-2 (pg/ml)	21,653 [11,185; 33,839]	31,916 [14,017; 36,448]	20,661 [10,953; 31,680]	0.241
Creatinine (mg/dl)	1.9 [1.5; 2.5]	1.9 [1.1; 2.3]	1.8 [1.5; 2.5]	0.56
Urea (mg/dl)	75 [48; 100]	38 [30; 63]	86 [57; 100]	**0.034**
Lactate (mmol/l)	2.3 [1.7; 6.7]	5.3 [2.3; 9.7]	2.0 [1.7; 4.2]	0.181
IL-6 (pg/ml)	678 [67; 5607]	10,092 [2520; 27,383]	83 [51; 654]	**< 0.001**
CRP (mg/dl)	8.8 [4.0; 15.9]	26 [3.2; 36]	7.4 [4.7; 12]	0.312
LDH (U/l)	1338 [554; 1940]	1257 [523; 1749]	1370 [660; 1962]	0.56
Bilirubin (mg/dl)	3,2 [1,5; 8,4]	3.2 [0.8; 3.5]	3.1 [1.7; 10.8]	0.525
Myoglobin (ng/ml)	17,130 [8400; 89,741]	7700 [894; 12,791]	44,666 [11,985; 106,009]	**0.018**
Creatinkinase (U/l)	4761.5 [840; 20,226.5]	1628 [97; 22,000]	14,466 [1363; 22,000]	0.063
Clinical parameters shortly before initiation of cytosorb				
Norepinephrine dosage (µg/kg/min)	0.24 [0.11; 0.41]	0.38 [0.27; 0.62]	0.15 [0.07; 0.38]	**0.039**
Fluid balance on day of CS treatment (ml/24 h)	2615 [1022; 5720]	6063 [3377; 7773]	1173 [464; 4350]	**0.011**

BMI, body mass index; SAPS-II, Simplified Acute Physiology Score; CVVHD(F), continuous veno-venous hemodialysis/hemodiafiltration; LDH, lactate dehydrogenase; CRP, C-reactive protein.

### Ang-2 plasma concentrations before, during and at the end of CS-application

The median (IQR) Ang-2 plasma concentrations for the whole cohort before initiation and after 6 and 12 h were 21,653 pg/ml (11,185; 33,839 pg/ml), 23,699 pg/ml (11,385; 34,801 pg/ml) and 17,732 pg/ml (10,475; 35,233 pg/ml), respectively. The median (IQR) Ang-2 plasma concentrations for the patients with septic shock at the defined timepoints were 31,916 pg/ml (14,017; 36,448 pg/ml), 35,254 (12,792; 36,231 pg/ml) and 35,742 pg/ml (20,748; 36,374 pg/ml), respectively. Further, the median (IQR) Ang-2 plasma concentration for the patients with rhabdomyolysis to the before mentioned timepoints were 20,661 pg/ml (10,953; 31,680), 17,560 pg/ml (11,060; 25,527 pg/ml) and 15,655 pg/ml (8768; 30,699 pg/ml), respectively.

**Fig. 1:**
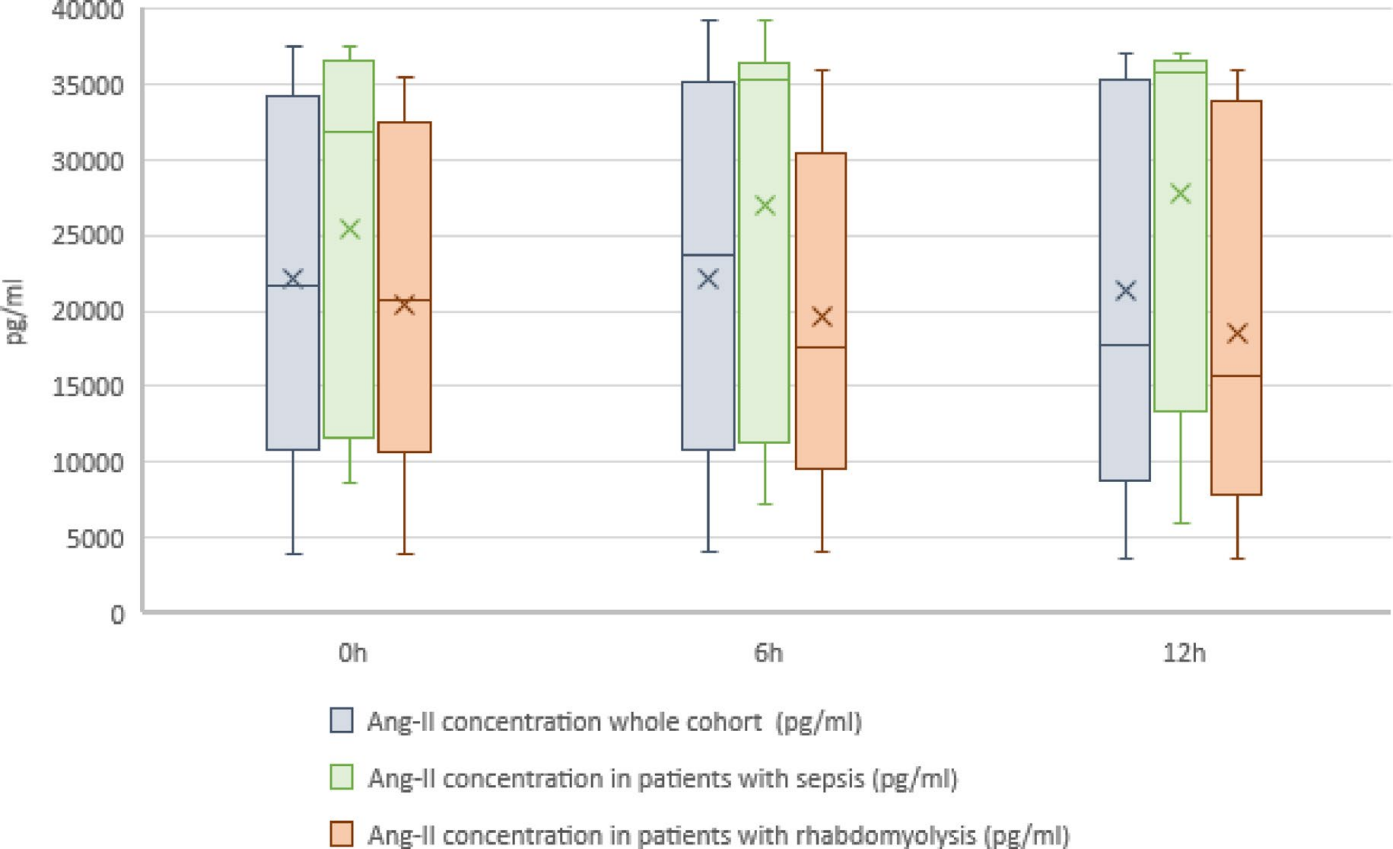
Plasma concentrations of Ang-2 at defined time points (whole cohort and subgroups). The boxes of the boxplots represent the interquartile-range (IQR) and the line the median. Whiskers were limited to 1.5 times the IQR. The cross represents the mean.

There was neither a significant difference in the plasma concentrations between the time points (before initiation vs. 6 h, before initiation vs. 12 h, 6 h vs. 12 h), nor between the subgroups. Figure 1 displays the plasma concentrations of Ang-2 before, during, and at the end of CS application in the whole population as well as in both subgroups.

### Extracorporeal elimination of Ang-2 with the adsorber cytosorb

**Fig. 2:**
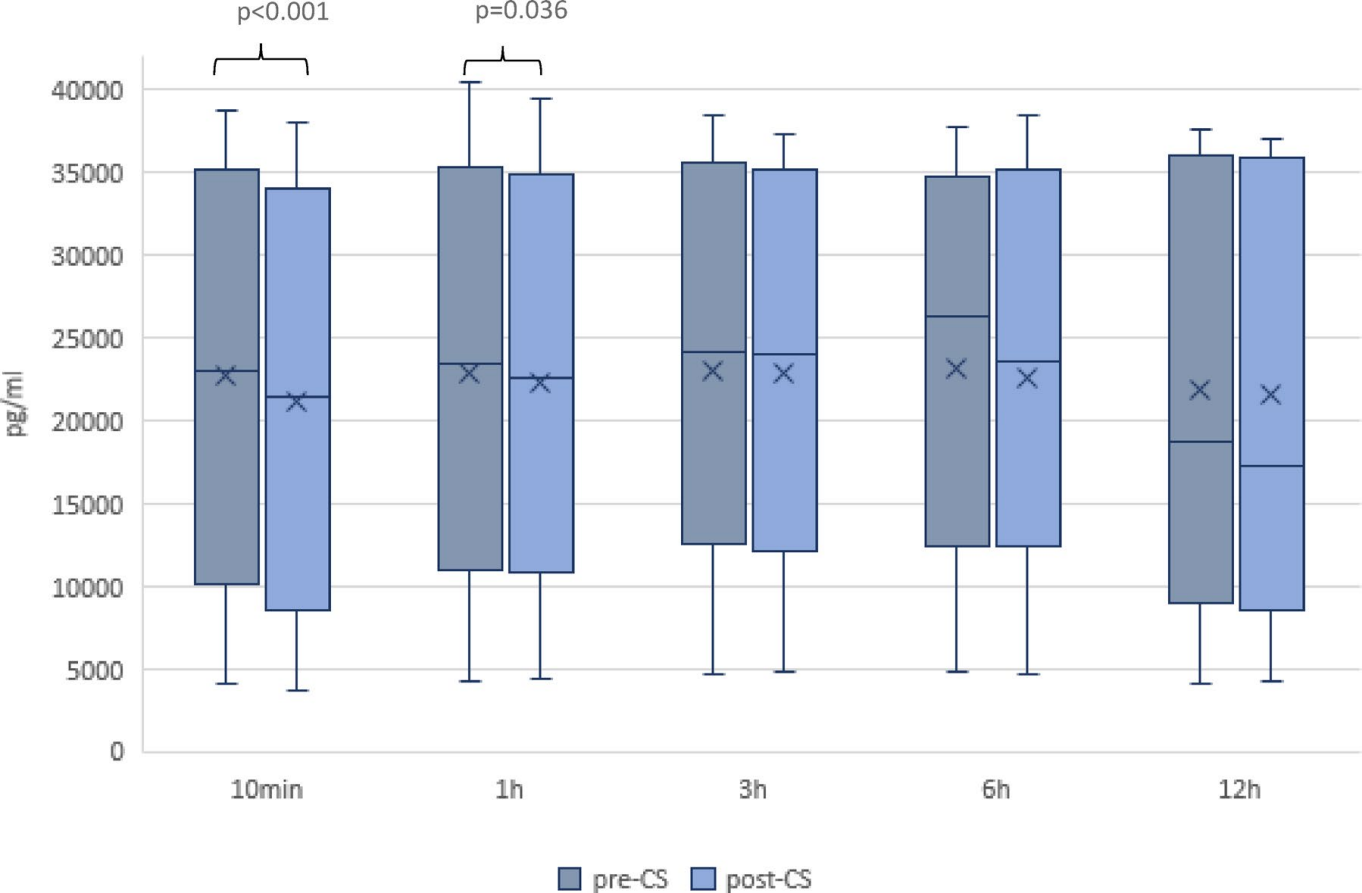
Extracorporeal concentrations of Ang-2 at the defined time points pre- and post-cytosorb. Extracorporeal concentrations of Ang-2 pre- and post-CS. The boxes of the boxplots represent the interquartile-range (IQR) and the line the median. Whiskers were limited to 1.5 times the IQR. The cross represents the mean.

A significant (*p* < 0.05) extracorporeal reduction of Ang-2 was observed at two time points (ten minutes and one hour) after the installation of CS. There was no significant extracorporeal reduction at any other time point. Figure 2 shows the extracorporeal concentrations of Ang-2 pre- and post CS. The median (IQR) extracorporeal clearance of Ang-2 ten minutes after the installation of CS was 8.2 ml/min (1.7; 11.1 ml/min). It decreased rapidly to 1.7 ml/min (− 1.5; 4.9 ml/min), 1.2 ml/min (− 0.5; 2.4 ml/min), 1.1 ml/min (− 1.1; 4.1 ml/min), and 1.3 ml/min (− 0.6; 2.3 ml/min) after 1, 3, 4, and 12 h, respectively.

### Fluid balance and catecholamine dosage before, during and after CS application

**Fig. 3:**
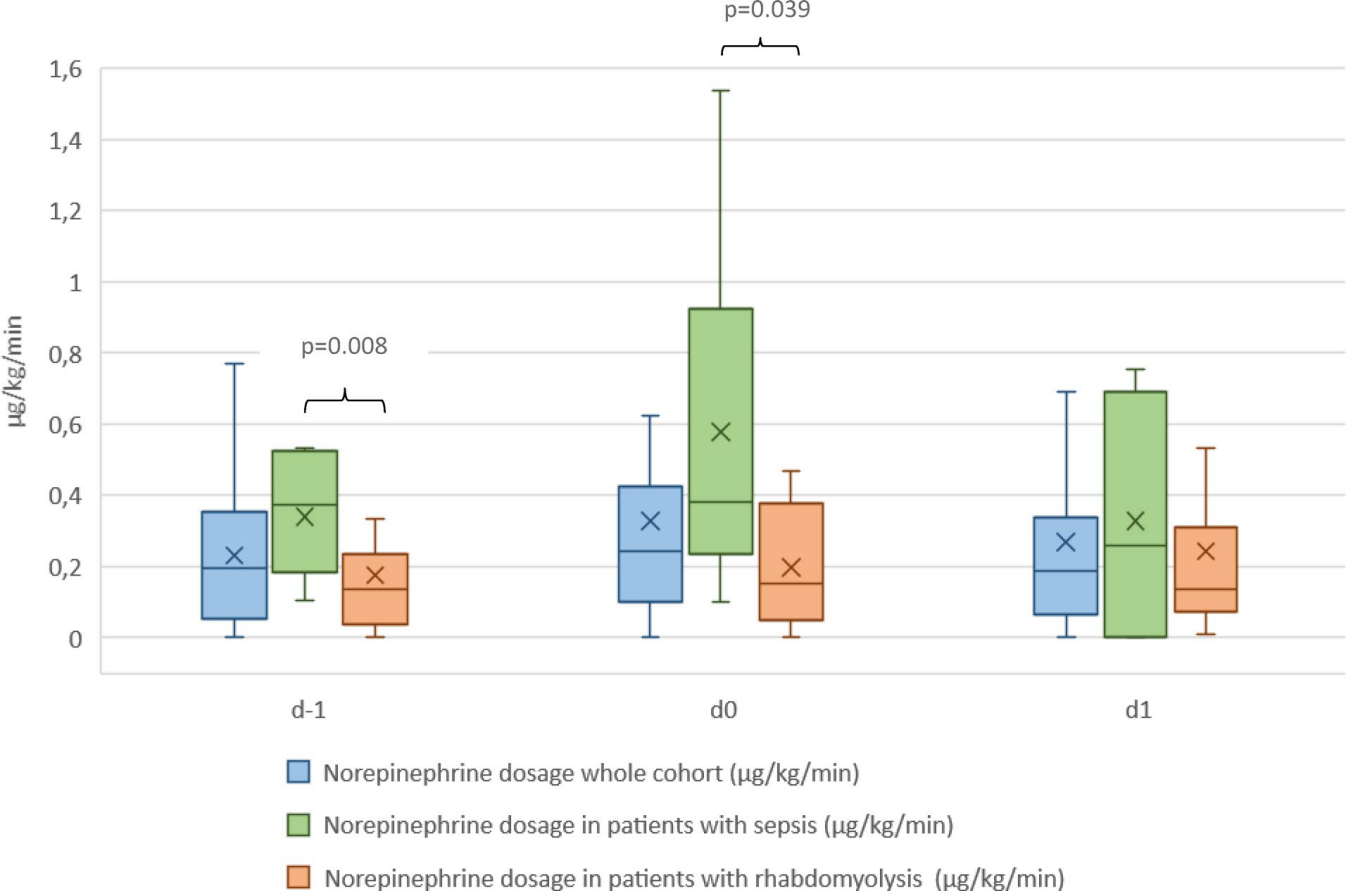
Norepinephrine dosages at specific time points in the whole cohort and subgroup. The boxes of the boxplots represent the interquartile-range (IQR) and the line the median. Whiskers were limited to 1.5 times the IQR. The cross represents the mean. d − 1 = 24 h prior to application, d0 = shortly before application, d1 = 24 h hours after starting CS therapy.

**Fig. 4:**
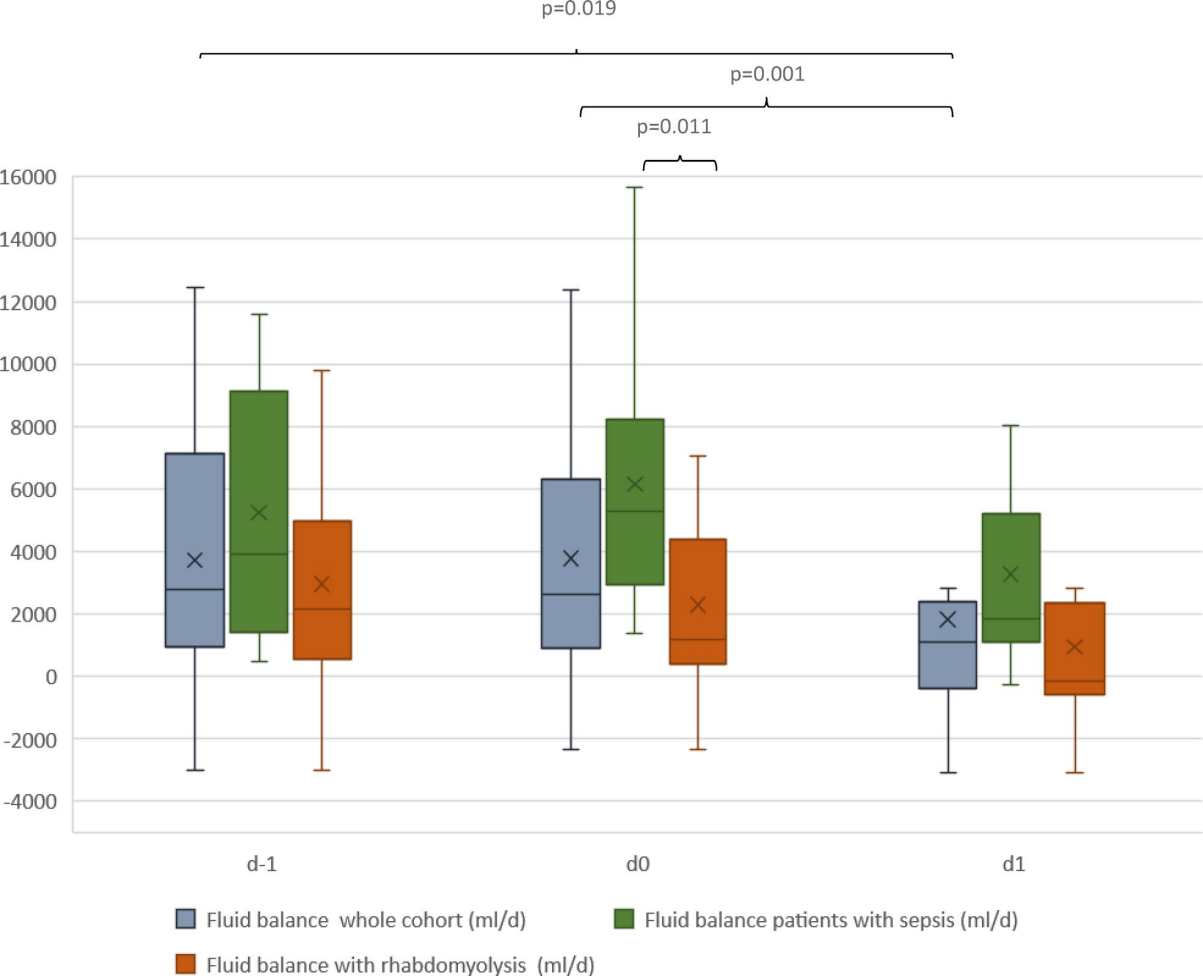
Fluid balance at specific time points in the whole cohort and subgroup. The boxes of the boxplots represent the interquartile-range (IQR) and the line the median. Whiskers were limited to 1.5 times the IQR. The cross represents the mean. d – 1 = 24 h prior to application, d0 = shortly before application, d1 = 24 h hours after starting CS therapy.

Fluid balance and catecholamine dosage as measurable parameters for increased vascular permeability were evaluated 24 h prior to application (d-1), shortly before application (d0), and 24 h hours after starting CS therapy (d1). The median (IQR) norepinephrine dosage was 0.20 (0.07; 0.33) µg/kg/min, 0.24 (0.11; 0.41) µg/kg/min and 0.19 (0.08; 0.33) µg/kg/min with no significant change during application. The median (IQR) fluid balance at d-1 was + 2791 ml (+ 1189; +6660 ml), at d0 + 2615 ml (+ 1022; +5720), and at d1 + 1113 ml (− 390; + 2372 ml), respectively. The patients’ fluid balance was significantly lower at d1 than d-1 (*p* = 0.019) or at d0 (*p* = 0.001). When comparing the two subgroups, the patients with septic shock had significantly higher norepinephrine doses at d−1 (*p* = 0.008) and at d0 (*p* = 0.039). Additionally, they had a significantly higher fluid balance at d0 (*p* = 0.011). Figures 3 and 4 display the norepinephrine dosages and fluid balances for the whole cohort and the subgroups.

There was no significant correlation between the Ang-2 concentration and the norepinephrine dosage at d0 (ρ = 0.404; *p* = 0.041) and the fluid balance at d0 (ρ = 0.238; *p* = 0.241). There was also no correlation between Ang-2 concentration and myoglobin (ρ = 0.044; *p* = 0.831) or IL-6 (ρ = 0.4; *p* = 0.043). Furthermore, Ang-2 did not correlate with the 28-days mortality (*r* = 0,333; *p* = 0.048).

## Discussion

Ang-2 plays a pivotal role in angiogenesis. Its association with several diseases as a biomarker and as a potential therapeutic approach has become increasingly important^3^. The only previously described method of extracorporeal removal is plasma exchange, as described by Lovric et al.^23^. Given that Ang-2, with a molecular size of 60–70 kDa, is at the upper limit of the CS adsorption spectrum, it seems plausible that extracorporeal removal may be possible. Moreover, there is evidence that hydrophilic molecules like bile acids or larger molecules, such as creatine kinase (80 kDa), can also be adsorbed, as CS adsorption is based on hydrophobic interactions^29–32^. Our results showed no statistically significant differences in plasma Ang-2 concentrations before, during and after CS application. A potential explanation for the observed lack of adsorption is that Ang-2 predominantly forms dimers, but also tetramers, which results in an increased molecular weight^3^. However, there was a significant but relatively small extracorporeal reduction in Ang-2 levels in the first hour following the application of CS. As previously mentioned, CS is capable of adsorbing a variety of molecules, including both hydrophobic molecules with a size of up to 60 kD and hydrophilic molecules, as well as molecules that are larger in size. Therefore, there is a possibility that CS was already fast saturated by other substrates, such as myoglobin, bilirubin, or cytokines, which have also been elevated in our patients^29,32,33^.

It has been demonstrated that high Ang-2 concentrations are associated with capillary leakage, frequently observed in patients with sepsis and septic shock^28^. Furthermore, it has been identified as a biomarker for mortality, fluid overload, and catecholamine requirement^9,11,14^. However, no correlation was observed between the Ang-2 concentrations prior to CS application and norepinephrine requirement, fluid balance, myoglobin, IL-6 and 28-day mortality. Several studies have indicated a correlation between Ang-2 and acute kidney injury. However, all of the patients in the study were already suffering from acute kidney injury, which required renal replacement therapy. Therefore, it is not possible to draw any conclusions regarding this association^9,13–16,29^.

A significantly lower fluid balance on the day following CS suggests a lower volume requirement for the patients. Ang-2 has been demonstrated to lead to endothelial disruption and increased vascular permeability. Consequently, the removal of Ang-2 could potentially lead to improved hemodynamic stabilization^3^. Given that plasma Ang-2 levels remained relatively stable throughout the course of CS, alternative explanations appear more probable. It is similarly conceivable that the appropriate administration of antibiotic therapy or surgical infection control may be responsible for the observed improvement in hemodynamic stability^28^.

We analyzed two subgroups based on the indication for CS therapy: septic shock and rhabdomyolysis. Comparing these groups, there was no significant difference between the initial Ang-2 concentrations. In contrast, patients with septic shock had higher norepinephrine doses and higher volume requirements, reflected by a higher fluid balance before and during CS therapy. Although both conditions allow for the administration of fluids, aggressive fluid resuscitation is crucial in septic shock, especially in the first hours^34^. However, the primary objective in rhabdomyolysis is to achieve high diuresis to prevent tubular obstruction and facilitate myoglobin clearance^35^. Therefore, it is likely that these patients will have a less positive fluid balance than patients with septic shock. The patients with septic shock exhibited significantly elevated IL-6 levels, while those with rhabdomyolysis demonstrated significantly elevated myoglobin levels. These findings align with the clinical characteristics of both conditions^28,29^.

Patients with rhabdomyolysis exhibited Ang-2 levels that were statistically equivalent to those observed in patients with septic shock. To date, no such association between Ang-2 and rhabdomyolysis has been described in the literature. Nevertheless, severe rhabdomyolysis in critically ill patients can also result in vasoplegia and capillary leakage, which may be associated with elevated Ang-2 concentrations^31^. Our data suggest that Ang-2 may serve as a non-specific biomarker of endothelial dysfunction in critically ill patients, regardless of its origin. Various authors have described increased concentrations of Ang-2 and a prognostic correlation in intensive care patients, e.g. in the context of sepsis^10,11^, renal failure^15,17^, ARDS^30,36^, and acute liver dysfunction^37^. Since most studies are limited to one disease entity, a simultaneous evaluation of the role of Ang-2 in different diseases would be desirable in the future.

In conclusion, the CS adsorber cannot effectively remove Ang-2 and is therefore unsuitable for its removal from the body. Whether the reduced positive balance in patients after CS use is due to the adsorber itself or to other causal and supportive therapies cannot be answered within the scope of this study. Our data indicate that severe rhabdomyolysis is also associated with high Ang-2 concentrations. As all patients require dialysis, it would be beneficial in the future to evaluate patients with rhabdomyolysis without acute kidney injury and in particular renal replacement therapy.

This post-hoc analysis is subject to several limitations. Firstly, the number of patients included is relatively small. However, the primary question of the adsorption capacity can be answered with a high degree of reliability using this number of patients. Secondly, both CVVHD and CVVHDF were used as dialysis modalities, yet this should have no impact on the Ang-2 elimination of CS itself as the samples were collected right before and after CS. Furthermore, as there was no change in the plasma concentrations of Ang-2, the different dialysis modalities should not impact the clearance of Ang-2. Thirdly, Ang-2 concentrations were only measured over a period of 12 h, with no long-term data available. This is a desirable future prospect, as it would allow for a more comprehensive assessment of the relationship between Ang-2 and outcome.

## Conclusion

The Cytosorb adsorber is not suitable for the extracorporeal removal of the endothelial biomarker Ang-2. Therefore, Ang-2 can still be considered a reliable biomarker for endothelial dysfunction even in the presence of Cytosorb therapy in critically ill patients. Further studies should be conducted to determine whether the removal of Ang-2 could be beneficial when utilizing an appropriate device. Furthermore, the role of Ang-2 in patients with rhabdomyolysis requires further investigation.

## Data Availability

All data generated or analyzed during this study are included in this published article.

## Abbreviations

AKI: Acute kidney injury
Ang-1: Angiopoietin-1
Ang-2: Angiopoietin-2
ARDS: Acute respiratory distress syndrome
BMI: Body mass index
CRP: C-reactive protein
CS: Cytosorb
CVVHD(F): Continuous veno-venous hemodialysis/hemodiafiltration
EDTA: Ethylenediaminetetraacetic acid
ICU: Intensive care unit
IL-6: Interleukin-6
LDH: Lactate dehydrogenase
RRT: Renal replacement therapy
SAPS II: Simplified Acute Physiology Score
